# Novel insights into molecular patterns of *ROS1* fusions in a large Chinese NSCLC cohort: a multicenter study

**DOI:** 10.1002/1878-0261.13509

**Published:** 2023-08-28

**Authors:** Shengyu Zhou, Fayan Zhang, Mengxiang Xu, Lei Zhang, Zhengchuang Liu, Qiong Yang, Chunyang Wang, Baoming Wang, Tonghui Ma, Jiao Feng

**Affiliations:** ^1^ Clinical Nursing Department, School of Nursing and Rehabilitation, Cheeloo College of Medicine Shandong University Jinan China; ^2^ Department of Respiratory and Critical Care Medicine, Qilu Hospital, Cheeloo College of Medicine Shandong University Jinan China; ^3^ College of Traditional Chinese Medicine Shandong University of Traditional Chinese Medicine Jinan China; ^4^ Jichenjunchuang Clinical Laboratory Hangzhou China; ^5^ Genecn‐Biotech Co.Ltd Hangzhou China; ^6^ Cancer Center, Daping Hospital Army Medical University Chongqing China; ^7^ Key Laboratory of Gastroenterology of Zhejiang Province Zhejiang Provincial People's Hospital, People's Hospital of Hangzhou Medical College China; ^8^ General Surgery, Cancer Center Zhejiang Provincial People's Hospital (Affiliated People's Hospital, Hangzhou Medical College) China; ^9^ Cancer Center, Key Laboratory of Tumor Molecular Diagnosis and Individualized Medicine of Zhejiang Province Zhejiang Provincial People's Hospital, Affiliated People's Hospital, Hangzhou Medical College China; ^10^ General Surgery, Department of Gastrointestinal and Pancreatic Surgery, Cancer Center Zhejiang Provincial People's Hospital, Affiliated People's Hospital, Hangzhou Medical College China; ^11^ School of Pharmacy Hangzhou Normal University China

**Keywords:** Chinese, DNA‐NGS, NSCLC, RNA‐NGS, *ROS1*

## Abstract

ROS proto‐oncogene 1, receptor tyrosine kinase (*ROS1*) rearrangements are a crucial therapeutic target in non‐small cell lung cancer (NSCLC). However, there is limited comprehensive analysis of the molecular patterns of *ROS1* fusions. This study aimed to address this gap by analysing 135 *ROS1* fusions from 134 Chinese NSCLC patients using next‐generation sequencing (NGS). The fusions were categorized into common and uncommon based on their incidence. Our study revealed, for the first time, a unique distribution preference of breakpoints within *ROS1*, with common fusions occurring in introns 31–33 and uncommon fusions occurring in introns 34 and 35. Additionally, we identified previously unknown breakpoints within intron 28 of *ROS1*. Furthermore, we identified a close association between the distribution patterns of fusion partners and breakpoints on *ROS1*, providing important insights into the molecular landscape of *ROS1* fusions. We also confirmed the presence of inconsistent breakpoints in *ROS1* fusions between DNA‐based NGS and RNA‐based NGS through rigorous validation methods. These inconsistencies were attributed to alternative splicing resulting in out‐of‐frame or exonic *ROS1* fusions. These findings significantly contribute to our understanding of the molecular characteristics of *ROS1* fusions, which have implications for panel design and the treatment of NSCLC patients with *ROS1* rearrangements.

## Graphical abstract

Diverse genomic breakpoints of *ROS1* rearrangements were identified by DNA‐based next‐generation sequencing (NGS) in non‐small cell lung cancer. Unique distribution preference between different *ROS1* fusions were investigated. Sequencing revealed a high amount of rare *ROS1* fusions in introns 34 and 35 of *ROS1*. RNA‐NGS is suggested to confirm functional *ROS1* fusions from DNA sequencing, as inconsistent results of uncommon fusions between DNA‐NGS and RNA‐NGS were obtained.

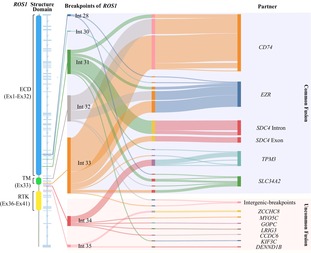

**Abbreviations**
*ALK*
: ALK receptor tyrosine kinase
*CCDC30*
: coiled‐coil domain containing 30
*CCDC6*
: coiled‐coil domain containing 6
*CD74*
: CD74 molecule
*DENND1B*
: DENN domain containing 1B
DNA NGS: DNA‐based next‐generation sequencing
*EYS*
: eyes shut homolog
*EZR*
: ezrin
FFPE: formalin‐fixed paraffin‐embedded
FISH: fluorescence *in situ* hybridization
*GOPC*
: Golgi associated PDZ and coiled‐coil motif containing
*KIF3C*
: kinesin family member 3C
*LRIG3*
: leucine‐rich repeats and immunoglobulin like domains 3
MSKCC: Memorial Sloan Kettering Cancer Center
*MYH9*
: myosin heavy chain 9
*MYO5C*
: myosin VC
NGS: next‐generation sequencing
NSCLC: non‐small cell lung cancer
*PWWP2A*
: PWWP domain containing 2A
*RET*
: RET proto‐oncogene
RNA NGS: RNA‐based next‐generation sequencing
*ROS1*
: ROS proto‐oncogene 1
RTK: receptor tyrosine kinase
RT‐PCR: reverse transcription polymerase chain reaction
*SDC4*
: syndecan 4
*SLC34A2*
: solute carrier family 34 member 2
*TCOF1*
: treacle ribosome biogenesis factor 1
*TFG*
: trafficking from ER to Golgi regulator
TKI: tyrosine kinase inhibitor
*TPM3*
: tropomyosin 3
*ZCCHC8*
: zinc finger CCHC‐type containing 8
*ZNF158*
: zinc finger protein 158

## 1 Introduction

Lung cancer, particularly non‐small cell lung cancer (NSCLC), has emerged as a major global health threat. In this context, it is crucial to accurately detect *ROS1* fusions, as they are present in up to 20–30% of NSCLC patients with targetable rearrangements [1]. Unlike traditional chemotherapy and immunotherapy, which yield limited responses, patients with *ROS1* fusions have shown significant improvements in survival and quality of life through the use of tyrosine kinase inhibitors (TKIs) such as Crizotinib, Repotrectinib, and Entrectinib [2, 3, 4, 5, 6]. Therefore, the precise detection of *ROS1* fusions can have a profound impact on the outcomes of advanced NSCLC patients, providing them with substantial benefits.

In the past few decades, several methods have been developed and established for detecting fusions. Among them, fluorescence *in situ* hybridization (FISH) and reverse transcription polymerase chain reaction (RT‐PCR) are traditional methods for *ROS1* fusions detection due to the low cost and handle‐ability [7, 8]. However, the utility of those methods were limited by the low‐throughput, especially for unknown *ROS1* fusions which were never reported before [9]. Moreover, the whole genome dispersedly distributed fusion partners and widely spanned breakpoints easily lead to missed detection of *ROS1* fusions by these two conventional techniques [10]. Beyond that, previous studies have reported that different *ROS1* fusion variants respond differently to *ROS1* inhibitors [11, 12]. Therefore, it is vitally important for clinicians to identify targetable *ROS1* fusions for developing effective therapeutic strategies in NSCLC patients.

With the advancements in molecular biology technologies, next‐generation sequencing (NGS) has become a standard clinical diagnostic method for identifying a wide range of fusion events in NSCLC with high throughput [13]. Among which, DNA‐based next‐generation sequencing (DNA NGS) enables simultaneously identifying fusion partners and genomic breakpoints from hundreds of gene [14]. However, accurately detecting *ROS1* fusions using DNA NGS poses significant challenges due to the highly dispersed distribution of fusion breakpoints and the complex structures within the introns of the *ROS1* gene. Previous studies have found that *ROS1* fusion was the most frequently missed receptor tyrosine kinase (RTK) rearrangement events by DNA NGS assays [15, 16]. One reason for this was the *ROS1* intron 31 contains numerous repetitive elements that are distributed throughout the genome, making it difficult to map the reads accurately [16]. Additionally, the genomic fusions identified by DNA sequencing may not always predict the presence of functional transcripts due to the intricate transcriptional processes involved [15]. For instance, intergenic‐breakpoint *ROS1* rearrangements theoretically should not be transcribed due to the lack of a promoter, but studies have showed some of them can unequivocally produce functional fusion transcripts [17]. Besides that, the functional implications of rare fusions identified at the genomic level are not easily predictable [18, 19]. Therefore, validation of genomic fusions at transcriptional level was critical to optimize treatment strategy. In comparison to DNA NGS, RNA‐based NGS (RNA NGS) offers superior detection of functional fusions as it specifically sequences the coding regions rather than the introns, enabling more accurate identification of functional *ROS1* fusions [10, 19]. However, the high dependence on RNA quality and the substantial sample amount requirement have limited the widespread use of RNA NGS, particularly in formalin‐fixed paraffin‐embedded (FFPE) samples, which are predominate sample type of NSCLC in clinical practice [20]. Therefore, finding ways to optimize the utility of both DNA NGS and RNA NGS in detecting functional *ROS1* fusions is essential.

In this study, we aimed to investigate the molecular landscape of *ROS1* fusions in a large cohort of Chinese NSCLC patients by integrating DNA and RNA sequencing. By doing so, we aimed to gain a comprehensive understanding of the characteristics of *ROS1* rearrangements in NSCLC. Our findings not only contribute to the knowledge about *ROS1* fusions in the Chinese population but also provide valuable insights into the detection capabilities of NGS for identifying *ROS1* fusions. These insights have the potential to improve clinical outcomes for NSCLC patients by facilitating accurate detection and targeted treatment of *ROS1* rearrangements.

## 2 Materials and methods

### 2.1 Patients and samples

Data of a Chinese cohort from multi‐center hospitals involving 134 NSCLC patients who were detected *ROS1* rearrangement positive by DNA NGS from January 2017 to December 2021 were retrospectively analysed (Table S1). In this study, only the 3′ *ROS1* rearrangements were targeted. Clinical samples were obtained from multiple centers. This study was approved by the ethical committee in Zhejiang Provincial People's Hospital (no. QT2022218), in accordance with the Declaration of Helsinki. The experiments were undertaken with the understanding and written consent of each subject. Here, *ALK*, *ROS1*, and *RET* fusion‐positive cancer patients from the MSK‐IMPACT Clinical Sequencing Cohort (MSKCC, CELL 2022) were used as the compared cohort [21]. The information of *ALK* and *RET* fusions in our cohort could be found in previous publications [22, 23].

### 2.2 DNA NGS

Genomic DNA was isolated from FFPE samples and detected using the previously disclosed targeted sequencing panel, which consists of all coding exons from 825 genes relevant to cancer and select introns from 44 commonly rearrangement genes, including *ROS1* intron31–35 [24]. TRIMMOMATIC (version 0.36) was used for quality control on the raw sequencing data to get rid of adapters and poor‐quality sections [25]. Using the BURROWS‐WHEELER ALIGNER tool (version 0.7.10), read local alignments to the hg19 genome (GRch37) were performed [26]. STRELKA (version 1.0.7), MUTECT (version 1.1.4), and GENEFUSE (version 0.6.1) were used to retrieve somatic single nucleotide variants, somatic insertions and deletions, and structural variations, respectively [27, 28, 29]. Based on recommendations from the Exome Aggregation Consortium, the variations were filtered and eliminated if their population frequency was more than 0.1% [30]. The remaining variations were annotated by Oncotator and Vep [31, 32].

### 2.3 RNA NGS

RNA was extracted using an AllPrep DNA/RNA FFPE Kit (Qiagen, Hilden, Germany) from FFPE samples. Using a Qubit RNA HS assay (Thermo Fisher Scientific, Waltham, MA, USA), the amount and quality of extracted RNA were quantified. Following strand‐specific cDNA synthesis with SuperScript III Reverse Transcriptase (Thermo Fisher Scientific, Waltham, MA, USA), cDNA libraries were constructed and hybridized with capture probe baits from the Fusioncapture panel, which contained the full transcripts of 395 cancer‐related common fusion genes [24]. The prepared libraries were subjected to paired‐end sequencing on the Illumina NovaSeq 6000 system (San Diego, CA, USA). Using Hisat2‐2.0.5, sequencing reads were mapped to the human reference genome (hg19) [33]. FusionMap was used to recognize gene fusions [34]. At least four unique pairs of supporting readings spanning the breakpoints between the two partners were required to call a fusion.

### 2.4 Statistical analysis

Categorical variables were compared using the chi‐square test. Analyses and data presentation were undertaken using R (version 4.2.0) (R Foundation for Statistical Computing, Vienna, Austria). The rearrangements and fusions were illustrated using ribbon for visualization: https://genomeribbon.com/. A two‐sided *P* value < 0.05 was considered to be statistically significant.

## 3 Results

### 3.1 Molecular pattern of *ROS1* rearrangements

**Fig. 1:**
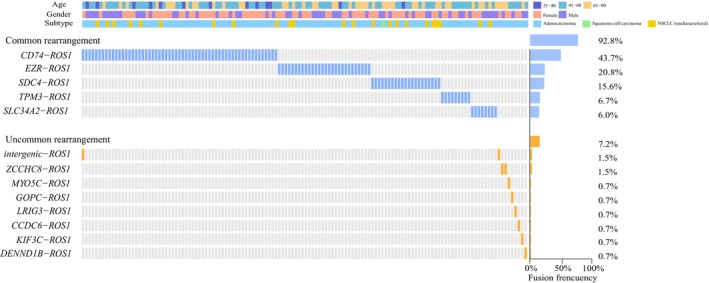
The molecular pattern of *ROS1* rearrangements in Chinese cohort. Oncoprint heatmap of 135 *ROS1* rearrangements from 134 NSCLC patients in local cohort. Each column represents a patient and each row represents a *ROS1* rearrangement. Blue column represents “Common Rearrangement”, Orange column represents “Uncommon Rearrangement”.

In this study, 135 *ROS1* fusions were identified in 134 NSCLC Chinese patients by DNA NGS and those fusions were classified as common and uncommon rearrangements based on the incidence (frequency limit = 5%). (Fig. 1, Table 1). Among the common rearrangements, *CD74*‐*ROS1* (43.7%, 59/135) was the most frequent, followed by *EZR*‐*ROS1* (20.8%, 28/135), *SDC4*‐*ROS1* (15.6%, 21/135), *TPM3*‐*ROS1* (6.7%, 9/135) and *SLC34A2‐ROS1* (6.0%, 8/135), respectively (Fig. 1). In the uncommon group, 8 *ROS1* fusions with different partners were identified. The most frequent among them were Intergenic‐breakpoint *ROS1* and *ZCCHC8‐ROS1*, each accounting for 1.5% (2/135). The remaining fusions, including *MYO5C‐ROS1*, *GOPC‐ROS1*, *LRIG3‐ROS1*, *CCDC6‐ROS1*, *KIF3C‐ROS1*, and *DENNDIB‐ROS1*, were observed only once (Fig. 1).

**Table 1:** Patterns of *ROS1* rearrangements in NSCLC patients by DNA NGS.

Rearrangement (no.)	Position1:Position2	Counts
Common rearrangements (125)		
*CD74‐ROS1* (59)	int6:int33	31
	int6:int32	17
	int7:int33	6
	int6:int31	4
	ex8:int33	1
*EZR‐ROS1* (28)	int10:int33	13
	int10:int32	10
	int10:int31	3
	int10:int28	1
	int10:int30	1
*SDC4‐ROS1* (21)	int2:int31	11
	ex5:int33	6
	int4:int31	4
*TPM3‐ROS1* (9)	int7:int34	5
	int7:int31	1
	int9:int34	1
	ex10:int33	1
	ex10:int34	1
*SLC34A2‐ROS1* (8)	ex13:int33	3
	ex13:int31	2
	int4:int28	1
	int4:int31	1
	ex13:int32	1
Uncommon rearrangements (10)		
*ZCCHC8‐ROS1* (2)	int2:int35	2
Intergenic‐breakpoint *ROS1* (2)	Downstream *TCOF1*:int33	1
	Downstream *TCOF1*:int32	1
*CCDC6‐ROS1*	int5:int34	1
*MYO5C‐ROS1*	int30:int34	1
*GOPC‐ROS1*	int4:int34	1
*LRIG3‐ROS1*	int15:int34	1
*DENND1B‐ROS1* ^a^	int2:int35	1
*KIF3C‐ROS1* ^a^	int5:int31	1

^a^ Novel rearrangement.

To assess the distribution characteristics of *ROS1* rearrangements in different populations, the largest published cancer genomic study of the MSKCC (Memorial Sloan Kettering Cancer Center) cohort was included in the analysis [21]. For *ROS1* common fusions, *CD74*‐*ROS1* (52.4%, 34/65), *EZR*‐*ROS1* (15.5%, 10/65), *SDC4*‐*ROS1* (13.8%, 9/65), *SLC34A2‐ROS1* (6.2%, 4/65) and *TPM3*‐*ROS1* (3.1%, 2/65) were distributed from high to low in the MSKCC cohort (Fig. S1a). In the uncommon group, 6 *ROS1* fusion types, including *LRIG3‐ROS1*, *CCDC6‐ROS1*, *CCDC30‐ROS1*, *MYH9‐ROS1*, *ZNF158‐ROS1* and *EYS‐ROS1* were identified, each representing 1.5% (1/65) (Fig. S1a).

Interestingly, although the types of common rearrangements were similar between our cohort and the MSKCC cohort, the incidence of *TPM3‐ROS1* was twice as high in our cohort compared to the MSKCC cohort (6.7% versus 3.1%) (Fig. 1, Fig. S1a, Tables S2 and S3). In the uncommon group, the dramatic difference in the type of *ROS1* rearrangement partners could be found between our and MSKCC cohort, with only 2 (*CCDC6* and *LRIG3*) out of 12 partners were identified in both cohorts, while the remaining partners were unique to each cohort (Fig. 1, Fig. S1a). Notably, we identified four novel *ROS1* rearrangements in our cohort, including two intergenic‐breakpoint *ROS1* rearrangements (case 17 and case 128), *DENND1B‐ROS1* (case 133), and *KIF3C‐ROS1* (case 134), which were not reported in the MSKCC cohort (Fig. S1b,c).

### 3.2 Characteristics of *ROS1* chromosomal rearrangements

**Fig. 2:**
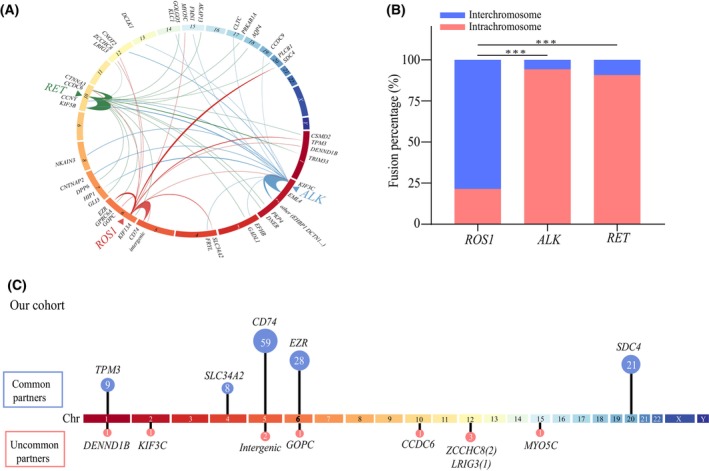
Characteristics of *ROS1* chromosomal rearrangements. (A) Circos plots graphically depicting *ROS1*, *ALK*, *RET* and their rearrangement partners in our cohort. (B) The histogram showed that ratio statistics of *ALK*, *RET* and *ROS1* chromosomal rearrangements in our cohort. Chi‐square test, ***P < 0.001. (C) Genome‐wide distribution patterns of *ROS1* common partners (top) and uncommon partners (bottom) in our cohort.

As shown in Fig. 2A,B, *ALK* and *RET* fusions in NSCLC mainly congregated intrachromosome (Table S4), however, *ROS1* rearrangements were significantly interchromosomal clustered (78.5%, 106/135). Interestingly, both common and uncommon partners of *ROS1* rearrangements displayed a diverse and distinct distribution patterns across the genome. The common *ROS1* rearrangement partners were observed on chromosome 1, 4, 5, 6 and 20. In the uncommon group, the eight different partners were distributed across seven different chromosomes, with the highest frequency on chromosome 12 (Fig. 2C).

Similar observations were made in the MSKCC cohort, where a significant difference was found in the major chromosomal distribution patterns between *ROS1* (interchromosomal) and *ALK/RET* (intrachromosomal) rearrangements (*P* < 0.001) (Fig. S2a,b). Regarding *ROS1* fusions, the common partners were located on chromosomes 1, 4, 5, 6, and 20. In contrast, uncommon partners were distributed across chromosomes 1, 6, 10, 12, 22, and X (Fig. S2c).

Although both cohorts exhibited a similar proportion of interchromosomal *ROS1* rearrangements, there were notable differences in the chromosome distribution of *ROS1* fusions between the two cohorts. Specifically, in comparison to our Chinese cohort, the uncommon *ROS1* fusion partners in the MSKCC cohort showed extensive dispersion across multiple chromosomes and lacked concentration.

### 3.3 Landscape of genomic breakpoints in *ROS1* fusions

**Fig. 3:**
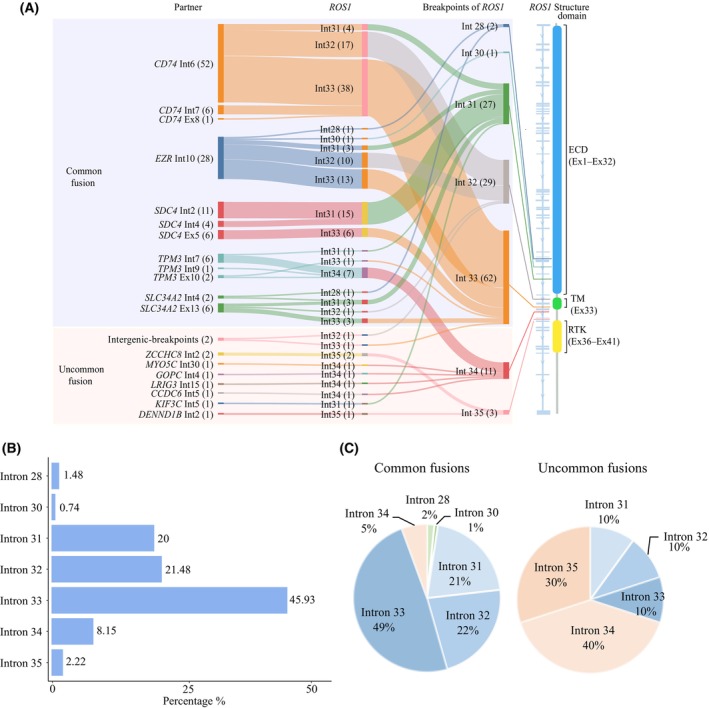
Distribution pattern of breakpoints in *ROS1* rearrangements. (A) Sankey diagram showing the flow of the detecting results on 135 *ROS1* rearrangements in 134 NSCLC patients according to DNA NGS by SankeyMATIC (http://sankeymatic.com/build/). From left to right, the first column showed fusion types and breakpoints of *ROS1* partners, whereas the middle column showed that breakpoints of *ROS1* in different rearrangement types. Finally, the right‐most column named Breakpoints of *ROS1*, categorized the location of *ROS1* breakpoints in different *ROS1* rearrangements. (B) Comparison bar chart showed the difference of *ROS1* breakpoints in local cohort. (C) The distribution of the *ROS1* breakpoints in common rearrangements and uncommon rearrangements. ECD, extra‐cellular domain; Ex, exon; Int, intron; RTK, receptor tyrosine kinase; TM, transmembrane domain.

Notably, distinct distribution pattern of *ROS1* breakpoints could be found between common and uncommon partners in our cohort. In the common group, the majority of *ROS1* breakpoints (87.4%, 118/135) were located in introns 31–33, with intron 33 showing significant enrichment (45.9%, 62/135) as the peak region. However, *TPM3‐ROS1*, in contrast to other common partners, predominantly exhibited breakpoints in a specific region of intron 34 (77.8%, 7/9) (Fig. 3A,B). In addition, intron 28 of *ROS1* was identified as an extra breakpoints domain for common *ROS1* fusions (Fig. 3A,B). Conversely, most of the breakpoints in the uncommon *ROS1* rearrangement group were primarily observed in introns 34–35 (70%, 7/10) (Fig. 3C).

A similar pattern of *ROS1* breakpoints characteristics among different *ROS1* fusions was also observed in the MSKCC cohort. In the MSKCC cohort, the breakpoints of common *ROS1* fusions were also mainly found in intron 33 (Fig. S3a–c). Additionally, the *ROS1* intron preference with different common partners was also variable. *CD74*, *SDC4*, *TPM3* and *SLC34A2* preferred fusing with intron 33, intron 31, intron 34 and intron 31 of *ROS1*, respectively (Fig. S3a). However, different from above‐mentioned partners with obvious preference, *EZR* displayed nearly the same preference in intron 32 and intron 33 of *ROS1* (Fig. S3a). In the uncommon *ROS1* rearrangement group, half of the breakpoints concentrated in intron 34, while the remaining breakpoints were distributed in intron 31, intron 33, and intron 35, respectively (Fig. S3b,c).

Interestingly, the specific breakpoint distribution characteristics were not only manifested among different *ROS1* fusion partners, but also within the same partner. For instance, in *SDC4‐ROS1* fusions, intronic‐breakpoints of *SDC4* were all fused with intron 31 of *ROS1* (Our cohort: 100%, 15/15; MSKCC cohort: 100%, 10/10), while all the *ROS1* breakpoints fused to *SDC4* exonic‐breakpoint were distributed in intron 33 (Our cohort: 100%, 6/6; MSKCC cohort: 100%, 1/1) (Fig. 3A, Fig. S3a).

### 3.4 Validation of *ROS1* common fusions by RNA NGS

**Fig. 4:**
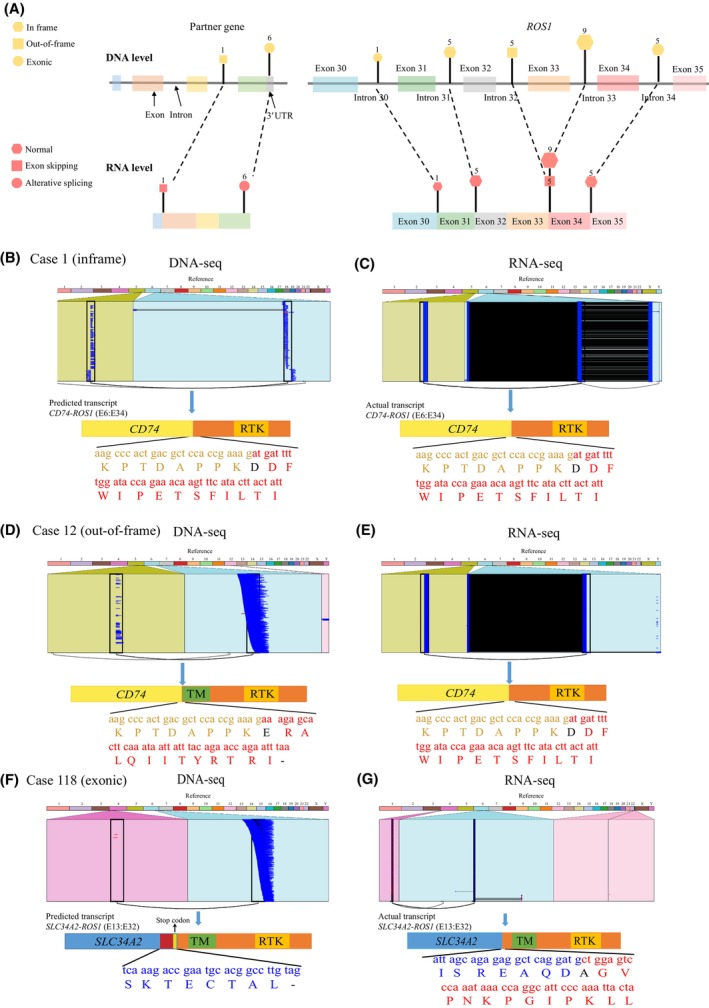
The strategy of alternative splicing for in‐frame transcription in *ROS1* common fusion. (A) Thirty‐two available samples harbouring *ROS1* common fusions detected by DNA NGS and RNA NGS. The upper part of the figure showed the predicted in‐frame transcripts, predicted out‐of‐frame transcripts and exonic transcripts at DNA level. The bottom part of the figure showed the normal transcripts, transcripts with exon‐skipping and transcripts with nucleotide deletion at RNA level. (B) DNA NGS ribbon picture and amino acid sequences of the predicted transcript in case 1. Black “D” was an amino acid formed after splicing. (C) RNA NGS ribbon picture and amino acid sequences of the actual transcript in case 1. Black “D” was an amino acid formed after splicing. (D) DNA NGS ribbon picture and amino acid sequences of the predicted transcript in case 12. Black “E” was an amino acid formed after splicing. “–” represented a stop codon. (E) RNA NGS ribbon picture and amino acid sequences of the actual transcript in case 12. Black “D” was an amino acid formed after splicing. (F) DNA NGS ribbon picture and amino acid sequences of the predicted transcript in case 118. “–” represented a stop codon. (G) RNA NGS ribbon picture and amino acid sequences of the actual transcript in case 118. Black “A” was an amino acid formed after splicing. RTK, receptor tyrosine kinase; TM, transmembrane domain.

To further validate whether *ROS1* fusions could transcribe to functional transcripts, 32 samples in the common group of our cohort with enough specimen were further validated by RNA NGS. Functional transcripts could be found in all samples, among which, 18 (56.3%, 18/32) *ROS1* fusions displayed consistent fusion structure at DNA and RNA level (Fig. 4A–C, Table S5). Sequence alignment results confirmed that all 18 *ROS1* fusions were in‐frame rearrangements (Case 1–8, 14–15, 17, 60–61, 88–89, 109–111, Fig. 4A–C, Table S5).

On the contrary, inconsistent results between DNA and RNA level were found in the rest 14 *ROS1* fusions (43.7%, 14/32) (Fig. 4A, Table S5). Among them, 8 cases (case 9–13, case 62–63, case 112) were out‐of‐frame genomic fusions at DNA level and the other 6 cases (case16, case 90, case 113, case 118–120) were exonic‐breakpoint *ROS1* fusions (Table S5). In the 8 out‐of‐frame *ROS1* fusions, breakpoint skipping could be found during transcription. Of them, the genomic breakpoints in 7 fusions were intron 32 of *ROS1*, which suggested the exon 33 would be involved at the transcript level. However, exon 34 was identified at the RNA level instead of exon 33 predicted by DNA sequencing (Case 9–13, 62–63, Fig. 4A,D,E, Table S5). Surprisingly, exon skipping was found in the partner gene in the last sample with out of frame (case 112, *TPM3‐ROS1*|E9:E35). Genomic breakpoint displayed in intron 9 of *TMP3*, indicating the involvement of *TPM3* exon 9 in the rearrangement at the transcript level. However, RNA NGS result revealed a *TPM3‐ROS1* fusion transcript joining *TPM3* exon 8 to *ROS1* exon 35 (Table S5). As for the 6 exonic‐breakpoint *ROS1* fusions, exonic breakpoints were all identified in the partners (Fig. 4A, Table S5). Among them, *SDC4‐ROS1* (Case 90) and *SLC34A2‐ROS1* (Case 118–120, Fig. 4F,G) fusion showed consistent fusion partners and breakpoints at DNA and RNA level. However, despite sharing the same fusion partner, *TPM3‐ROS1* (Case 113) and *CD74‐ROS1* (Case 16) fusion transcripts presented different breakpoints from those predicted from DNA sequencing.

In those *ROS1* fusions with inconsistent results between DNA and RNA level, alternative splicing was found to involve producing an in‐frame fusion transcript during the transcription process. For instance, a rearrangement of *SLC34A2* 3′UTR‐*ROS1* intron 31 was detected at the DNA level in case 118, and RNA NGS revealed that the *SLC34A2* mid‐exon 13, upstream of the predicted breakpoint detected by DNA NGS, fused to *ROS1* exon 32 (Fig. 4A,F,G).

### 3.5 Validation of *ROS1* uncommon fusions by RNA NGS

**Fig. 5:**
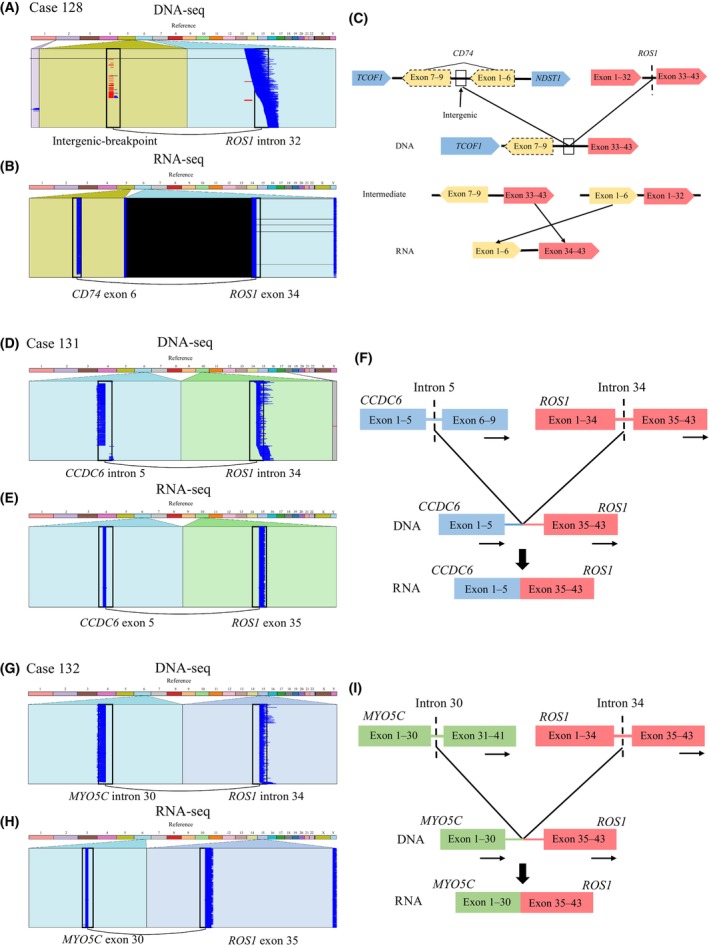
Genetic features and schematic diagram of uncommon fusion detected by DNA NGS and RNA NGS. (A) DNA NGS analysis of the intergenic‐breakpoint *ROS1* fusion in case 128. (B) RNA NGS analysis of *CD74‐ROS1* fusion. (C) Possible schematic diagram of intergenic‐breakpoint *ROS1* fusion detected by DNA NGS, but *CD74‐ROS1* fusion was identified by RNA NGS. (D) *CCDC6‐ROS1* rearrangement detected by DNA NGS in case 131. (E) *CCDC6‐ROS1* fusion identified by RNA NGS. (F) Schematic diagram of *CCDC6‐ROS1* fusion detected by DNA NGS and RNA NGS. (G) *MYO5C‐ROS1* rearrangement detected by DNA NGS in case 132. (H) *MYO5C‐ROS1* fusion identified by RNA NGS. (I) Schematic diagram of *MYO5C‐ROS1* fusion detected by DNA NGS, and RNA NGS.

Three uncommon *ROS1* rearrangements with enough specimen were successfully validated by RNA‐NGS (case 128, 131 and 132). In case 128, an intergenic‐breakpoint *ROS1* rearrangement transcribed to a *CD74‐ROS1* fusion transcript by joining *CD74* exon 6 to *ROS1* exon 34 at RNA level. Interestingly, the genomic breakpoint was located at downstream of *TCOF1* on the sense strand and in *CD74* intron 6 on the antisense strand, chromosomal inversion may lead to the inconsistent detection results between DNA and RNA sequencing (Fig. 5A–C). Beside the novel intergenic‐breakpoint *ROS1* fusion, previous reports shown that rare *ROS1* genomic rearrangements could transcribe to common fusion types or not produce functional fusion transcripts. However, in our study, two uncommon rearrangements, *CCDC6‐ROS1* and *MYO5C‐ROS1*, had identical fusion partners and breakpoints at both DNA and RNA level, perhaps owing to these two rare *ROS1* fusion forming via interchromosomal mechanism (Fig. 5D–I).

## 4 Discussion

Oncogenic drivers are gradually regarded as one of the main effectors in NSCLC, among which, *ROS1* fusions play a significant proportion [35]. To date, numerous TKIs had been approved to target *ROS1* rearrangements, however, variable clinical responses were observed in different *ROS1* fusions [36]. Therefore, profiling *ROS1* fusions in advanced NSCLC patients is essential to optimize treatment strategy. This study included 134 Chinese NSCLC patients with *ROS1* fusions, identifying diverse fusion partners, including novel partners *DENND1B* and *KIF3C*. Additionally, the type of fusion partners was found to have a significant impact on intron preference in *ROS1* in both our and MSKCC cohort. Common partners exhibited breakpoints mainly within introns 31–33, whereas rare *ROS1* fusions predominantly had breakpoints in introns 34 and 35. Moreover, functional transcripts were identified in all common *ROS1* fusions, but inconsistencies were observed between DNA NGS and RNA NGS for uncommon *ROS1* fusions. These findings provide valuable insights for optimizing the use of NGS in NSCLC patients with *ROS1* fusions.

Previous studies reported that different *ROS1* fusion partners determine the differential response and prognosis in clinic [11, 37, 38]. Therefore, comprehensive analysis of partner landscape of *ROS1* is essential. *ROS1* partners displayed considerable heterogeneity in different cancer types. For instance, Inflammatory myofibroblastic tumours are characterized by *TFG* as the main partner of *ROS1* [39]. While *PWWP2A* is the most common *ROS1* partner in Spitzoid neoplasms [40]. Beyond these, in adult gliomas, the main partner of *ROS1* fusions is *GOPC* [41]. However, *ROS1* fusion partners showed diverse pattern in NSCLC in both our and MSKCC cohort, with *CD74* being the most common partner, followed by *EZR*, *SDC4*, *TPM3*, and *SLC34A2*, indicating patients with *ROS1* fusions represent a heterogeneous NSCLC group. Furthermore, among the 16 rare fusions identified in both cohorts, only *LRIG3‐ROS1* and *CCDC6‐ROS1* were same to both. The high heterogeneity of partners may contribute to the variable efficacy of targeted therapies in different types of *ROS1* fusions [11, 12]. These findings highlighted the critical importance of comprehensive profiling of patients with *ROS1* fusions.

Optimizing breakpoint coverage is another critical aspect to enhance the fusion detection capability of DNA NGS. Most genomic breakpoints of oncogenic drivers distributed in intron, however, it is not feasible to achieve full coverage of all introns due to considerations of effectiveness and cost‐effectiveness [42, 43]. Therefore, careful selection of introns is essential in DNA NGS panel design. Our analysis of both cohorts revealed that the majority of breakpoints in common *ROS1* fusions were concentrated within introns 31–33. Furthermore, a unique distribution pattern for *TPM3‐ROS1* breakpoints was found within a specific region of intron 34. In contrast, breakpoints of uncommon *ROS1* fusions were mainly observed in introns 34–35, highlighting the variability in breakpoint distribution across different *ROS1* fusion events. These differences in breakpoint distribution have critical implications for the design of targeted NGS panels for *ROS1* fusion detection. Although the introns 28 and 30 were not covered by assay, breakpoints of *ROS1* fusions in introns 28 and 30 were firstly identified by our cohort. The successful detection of positive *ROS1* fusions in these patients attributed to the coverage of breakpoints of partners (*EZR* intron10 and *SLC34A2* intron 4) in the panel. This indicated the proportion of *ROS1* fusions happened in intron 28 and 30 might be underestimated. By incorporating these findings into the design of targeted NGS panels, the sensitivity and specificity of *ROS1* fusion detection assays can be significantly enhanced, which may lead to more precise diagnoses, enabling clinicians to make better‐informed treatment decisions for patients with *ROS1* fusion‐driven diseases.

Although DNA NGS has a broader mutation detection range compared to traditional methods like RT‐PCR and FISH [9], it may not always accurately represent the actual fusion transcripts due to complex transcriptional or post‐transcriptional processes, which can have implications for therapy strategies. For example, in our and previous studies [10], samples with genomic breakpoints in intron 32 of *ROS1*, the actual breakpoints were found to be located at exon 34 at RNA level rather than exon 33 as suggested by DNA NGS. Furthermore, based on the genomic structure of those *ROS1* fusion, the predicted transcript was found to be out of frame, suggesting that exon 33 might have been excluded through alternative splicing to generate a functional transcript. Interestingly, in common *ROS1* fusions, functional transcripts were found in 100% patients, indicating that DNA NGS detection is sufficient for common *ROS1* fusions identification in clinical testing. On the contrary, inconsistency result was found in rare *ROS1* fusions, such as in intergenic‐breakpoint *ROS1* fusions. Previous studies have demonstrated heterogeneous transcriptional outcomes for intergenic‐breakpoint rearrangements, where functional transcripts may or may not be generated. For example, as reported by Li et al [17], intergenic‐breakpoint *ROS1* fusions detected by DNA NGS were negative in RNA NGS. However, our cohort demonstrated intergenic‐breakpoint *ROS1* fusion detected by DNA NGS transformed to canonical *CD74*‐*ROS1* fusion, which is consistent with findings from another study [15]. These conflicting results highlight the importance of validation assays such as RNA NGS for cases harbouring genomic intergenic‐breakpoint *ROS1* rearrangements, to ensure the generation of an active chimeric protein. Moreover, unlike most of *ALK* and *RET* rare rearrangements which transformed into canonical fusions [22, 23]. The two rare fusions in this study, *CCDC6‐ROS1* and *MYO5C*‐*ROS1*, were entirely consistent at the DNA and RNA levels. This discrepancy may be attributed to the fact that most uncommon *ALK* and *RET* fusion partners reported by DNA NGS are in chromosomal proximity to *ALK* and *RET*, whereas *ROS1* fusion partners do not follow the same pattern [44].

Several limitations should be acknowledged in our study. First, due to the unavailability of specimens, some of the *ROS1* rearrangements identified by DNA NGS could not be validated by RNA NGS. However, the impact of this limitation is expected to be minimal, as different types of *ROS1* fusions were successfully validated by RNA NGS. Nevertheless, larger clinical studies are warranted to investigate whether various rare or intergenic‐breakpoint *ROS1* rearrangements can generate functional transcripts. Second, the complex structure of *ROS1* resulted in incomplete coverage of introns by the assay. Consequently, the initial screening approach in this cohort may have underestimated the proportion of true *ROS1* fusions. Future improvements in assay design and coverage may enhance the sensitivity of *ROS1* fusion detection. Furthermore, the lack of patient medication information prevented us from assessing the responses of different *ROS1* fusion subtypes to *ROS1* TKIs. Therefore, further studies are necessary to elucidate the associations between different *ROS1* fusion subtypes and clinical efficacy, which would provide valuable insights for clinical decision‐making.

## 5 Conclusions

In conclusion, our study provided a comprehensive profiling of *ROS1* fusion in Chinese NSCLC patients. Additionally, our findings suggest that DNA NGS alone is enough to be used as a reliable clinical test for common *ROS1* rearrangements. For rare *ROS1* rearrangements, clinical validation using both DNA NGS and RNA NGS is necessary to ensure accurate molecular diagnosis and effective *ROS1* inhibitor therapy. Our study highlights the importance of accurate detection and validation of *ROS1* fusions in clinical practice and paves the way for more effective targeted therapy for NSCLC patients.

## Conflict of interest

MX was employed by Jichenjunchuang Clinical Laboratory. CW, BW and TM were employed by Jichenjunchuang Clinical Laboratory and Genecn‐Biotech Co. Ltd. The authors declare no conflict of interest.

## Author contributions

SZ, FZ, TM and JF were involved in conception and design; MX and QY were involved in administrative support; QY, ZL, LZ and JF were involved in data collection and organization; MX, CW and BW finished the interpretation and visualization of results; SZ, MX, BW and CW wrote the original manuscript. All authors were involved in manuscript writing and revising and final approval of manuscript.

## Supporting information


**Fig. S1.** The molecular pattern of *ROS1* rearrangements in MSKCC cohort.
**Fig. S2.** Characteristics of *ROS1* chromosomal rearrangements.
**Fig. S3.** Distribution pattern of breakpoints in *ROS1* rearrangements.

**Table S1.** Characteristics of the ROS1 fusion‐positive Lung Cancer patients.
**Table S2.** Detail information of ALK/ROS1/RET rearrangments in 25 000 pts from MSKCC cohort.
**Table S3.** Comparison between local cohort and MSKCC for ROS1 fusion
**Table S4.** Comparison between intrachromosome and interchromosome of rearrangment in local cohort and MSKCC cohort.
**Table S5.** Detail information of ROS1 rearrangments in 134 patients by DNA‐based NGS.

## Data Availability

The data of our cohort presented in this study are available on request from the corresponding author. The data are not publicly available due to restrictions. Publicly available datasets were analysed in this study. This data can be found here: (MSKCC, CELL 2022).
