# Supplementary material for: Novel insights into molecular patterns of ROS1 fusions in a large Chinese NSCLC cohort: a multicenter study
Source: Mol Oncol. 2023 Aug 28;17(10):2200–12. doi: 10.1002/1878-0261.13509 (PMC10552890; doi:10.1002/1878-0261.13509)
Supplement: Supplementary file 1 — Fig. S1. The molecular pattern of ROS1 rearrangements in MSKCC cohort. Fig. S2. Characteristics of ROS1 chromosomal rearrangements. Fig. S3. Distribution pattern of breakpoints in ROS1 rearrangements. [file MOL2-17-2200-s001.pdf]

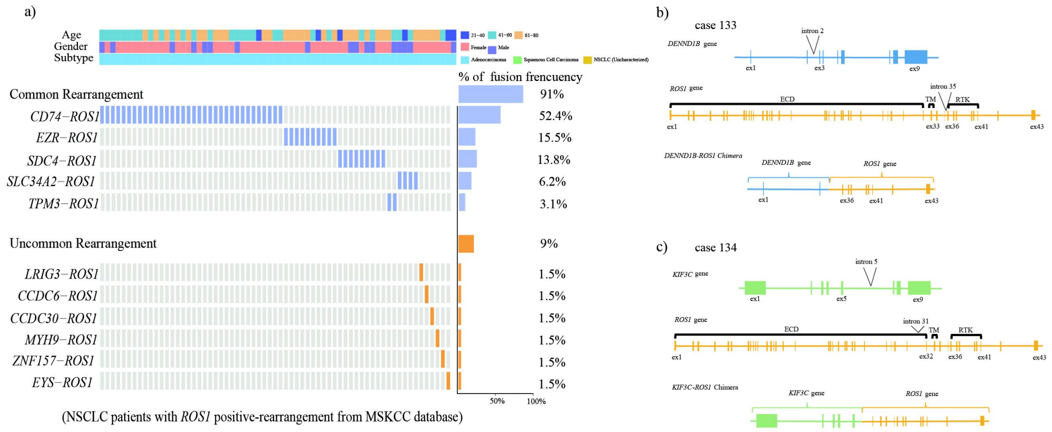

Figure S1 The molecular pattern of *ROS1* rearrangements in MSKCC cohort. a) Oncoprint heatmap of *ROS1* rearrangements in MSKCC cohort. Each column represents a patient and each row represents a *ROS1* rearrangement. Blue column represents "Common Rearrangement", Orange column represents "Uncommon Rearrangement". b) The structure of novel fusion in our cohort: *DENND1B-ROS1.* c) The structure of novel fusion in our cohort: *KIF3C-ROS1.*

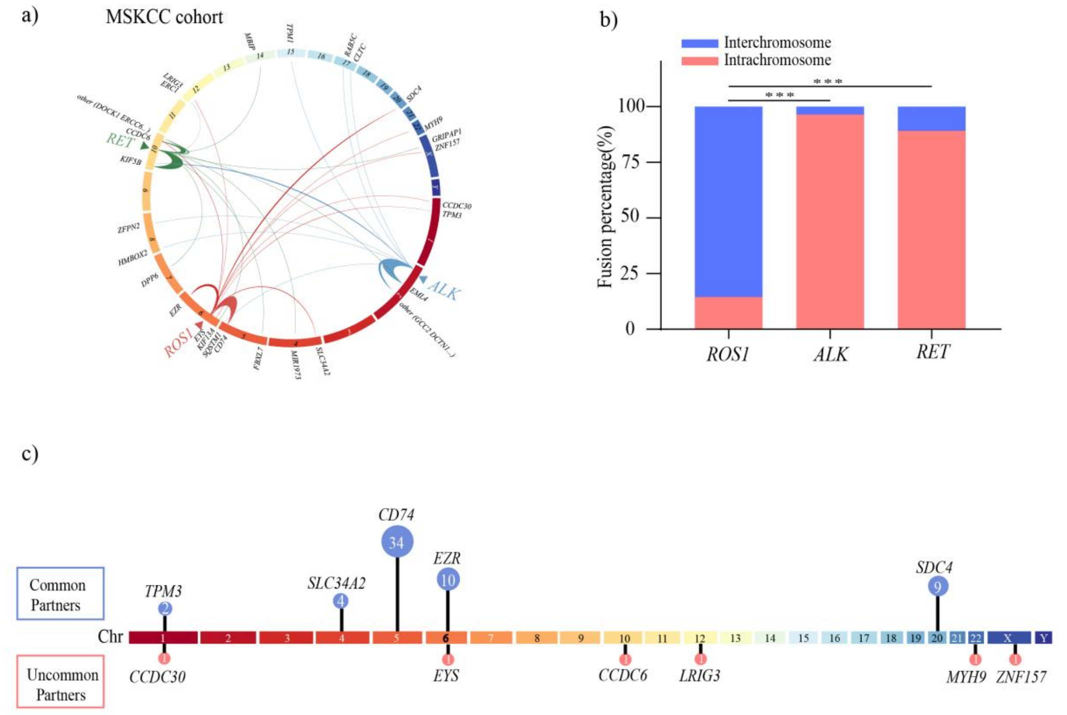

Figure S2. Characteristics of *ROS1* chromosomal rearrangements. a) Circos plots graphically depicting *ROS1*, *ALK*, *RET* and their rearrangement partners in MSKCC cohort. b) The histogram showed that ratio statistics of *ALK*, *RET* and *ROS1* chromosomal rearrangements in MSKCC cohort. *** $p < 0.001$. c) Genome-wide distribution patterns of *ROS1* common partners (top) and uncommon partners (bottom) in MSKCC cohort.

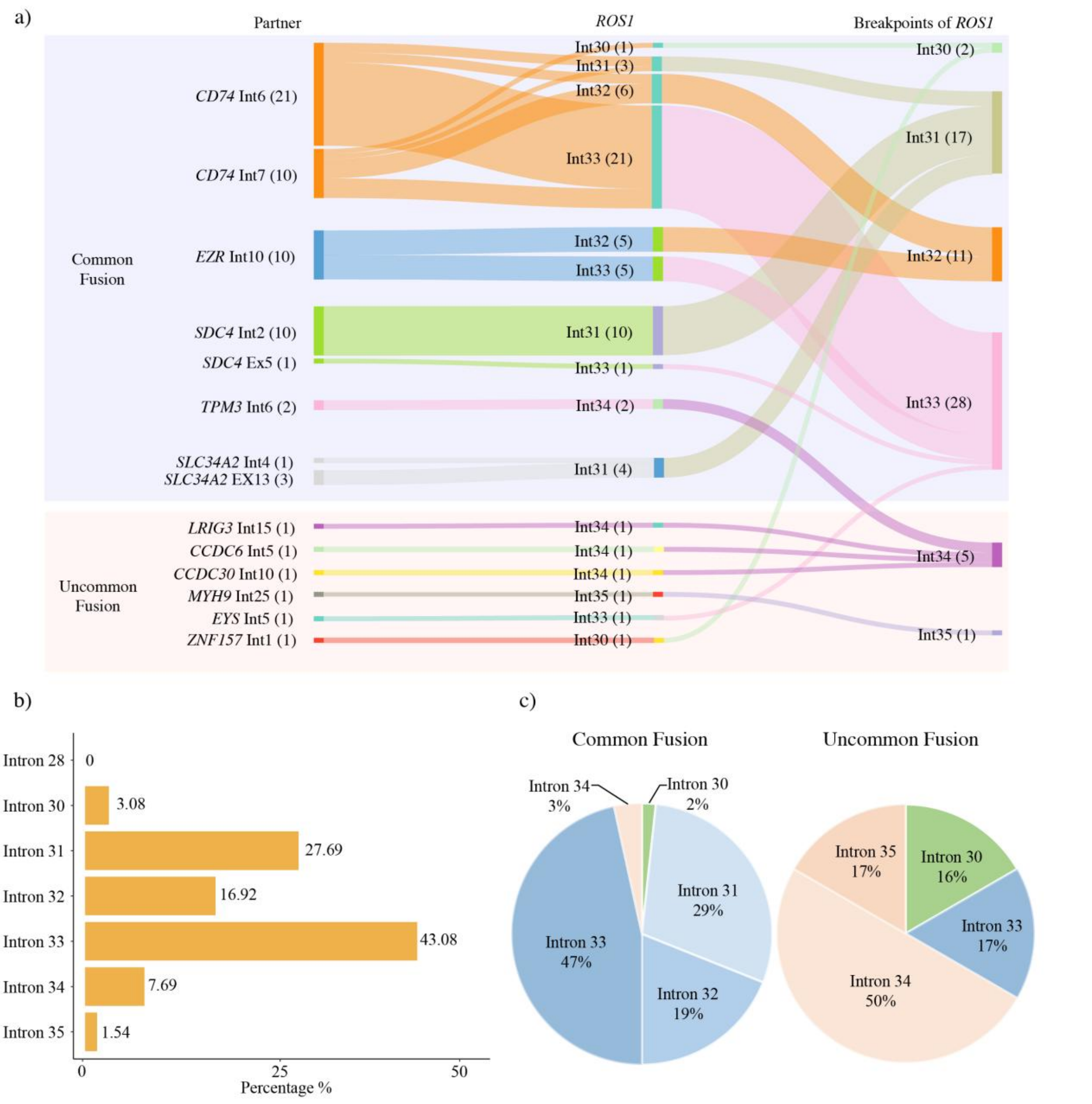

Figure S3. Distribution pattern of breakpoints in *ROS1* rearrangements. a) Sankey diagram showing the flow of the detecting results on *ROS1* rearrangements according to DNA NGS by SankeyMATIC (http://sankeymatic.com/build/). From left to right, the first column showed fusion types and breakpoints of *ROS1* partners, whereas the middle column showed that breakpoints of *ROS1* in different rearrangement types. Finally, the right-most column named Breakpoints of *ROS1*, categorized the location of *ROS1* breakpoints in different *ROS1* rearrangements. ECD: Extra-Cellular Domain; TM: Transmembrane Domain; RTK: Receptor Tyrosine Kinase. b) Comparison Bar Chart showed the difference of *ROS1* breakpoints in MSKCC cohort. c) The distribution of the *ROS1* breakpoints in common

rearrangements and uncommon rearrangements.
